# Genetic characterisation of Measles virus variants identified during a large epidemic in Milan, Italy, March–December 2017

**DOI:** 10.1017/S0950268818003606

**Published:** 2019-01-30

**Authors:** S. Bianchi, E.R. Frati, A. Lai, D. Colzani, G. Ciceri, M. Baggieri, A. Lamberti, S. Senatore, M. Faccini, F. Mazzilli, M. Gramegna, G. Zehender, F. Magurano, E. Tanzi, A. Amendola

**Affiliations:** 1 Department of Biomedical Sciences for Health, University of Milan, Milan, Italy; 2 Department of Biomedical and Clinical Sciences ‘Luigi Sacco’, University of Milan, Milan, Italy; 3 National Reference Laboratory for Measles and Rubella, Istituto Superiore di Sanità (ISS), Rome, Italy; 4 Health Protection Agency, Metropolitan Area of Milan, Milan, Italy; 5 DG Salute, UO Governo della prevenzione e tutela sanitaria, Lombardy Region, Milan, Italy; 6 Coordinated Research Center ‘EpiSoMI’, University of Milan, Milan, Italy

**Keywords:** Measles (rubeola), Surveillance, virus transmission pathways, autochthonous and imported variants

## Abstract

In 2017, Italy experienced a large measles epidemic with 5408 cases and four deaths. As Subnational Reference Laboratory of the Measles and Rubella surveillance NETwork (MoRoNET), the EpiSoMI (Epidemiology and Molecular Surveillance of Infections) Laboratory (University of Milan) set up rapid and active surveillance for the complete characterisation of the Measles virus (Mv) responsible for the large measles outbreak in Milan and surrounding areas (Lombardy, Northern Italy). The aims of this study were to describe the genetic profile of circulating viruses and to track the pathway of measles transmission. Molecular analysis was performed by sequencing the highly variable 450 nucleotides region of the N gene (N-450) of Mv genome. Two-hundred and ninety-nine strains of Mv were analysed. The phylogenetic analysis showed five different variants, two not previously described in the studied area, belonging to D8 and B3 genotypes. Three events of continuous transmission of autochthonous variants (D8-Osaka, D8-London and B3-Milan variants) and two events of continuous transmission of imported variants (B3-Dublin and D8-Hulu Langat) tracked five different transmission pathways. These pathways outlined two epidemic peaks: the first in April and the second in July 2017. The correlation between Mv variant and the epidemiological data may enable us to identify the sources of virus importation and recognise long-lasting virus transmission pathways.

## Introduction

The World Health Organization (WHO) planned to eliminate measles by 2015, but due to the numerous measles outbreak in the European Region in the last few years this did not occur [1] and consequently the WHO postponed the target date for the global eradication of measles to 2020 [2, 3]. Measles elimination depends mainly on achieving high vaccination coverage, closing immunity gaps and ensuring high-quality, case-based surveillance [4], which are the main goals of the European Region as outlined in the European Vaccine Action Plan 2015–2020 [5].

The process of verification of elimination will be based on documented evidence of the interruption of endemic measles transmission at national level [5]. Endemic transmission is defined as continuous transmission of an indigenous or imported Measles virus (Mv) for a period of 12 months or more in a defined geographical area. Measles is deemed to be eliminated when no endemic cases have been documented for a period of at least 12 months by a well-performing surveillance system [4].

Mv is a negative-sense, single-stranded RNA virus belonging to the *Paramyxoviridae* family, genus *Morbillivirus*. Sequencing of a 450 nt long fragment coding for the carboxyl-terminus of the Nucleoprotein (N-450) is currently recommended by the WHO for genotyping of Mv, since it provides the highest density of variable nucleotides within a relatively small sequence fragment [6]. The molecular characterisation of virus strains is essential for monitoring Mv transmission and the progress of elimination efforts [7]. The WHO recognises 24 genotypes of Mv [6, 8] and there are four predominant measles genotypes currently circulating worldwide: D8, B3, H1 and D4 [9]. Within genotypes, variants are defined as groups of viruses with identical or nearly identical N-450 sequence. Moreover, WHO refers to ‘named’ strains when identical strains are identified due to their prevalence within MeaNS (Measles Nucleotide Surveillance) database [6].

The correlation between genetic and epidemiological data may enable us to identify the sources of virus importation and recognise long-lasting virus transmission pathways.

In 2017, Italy experienced a large measles epidemic with 5408 cases and four deaths [10, 11]. Most cases occurred in Piedmont and Lombardy (Northern Italy), Tuscany, Lazio and Abruzzo (Central Italy) and Sicily (Southern Italy) and were older than 15 years (median age: 27 years). Overall, 88% of measles cases were not vaccinated, and 44% were hospitalised. Seventy-nine per cent of the cases were laboratory confirmed [11, 12]. From March 2017, as one of the two Subnational Reference Laboratories of the Measles and Rubella surveillance NETwork (MoRoNET) [13] in the Lombardy Region, EpiSoMI Laboratory set up rapid and active surveillance for the complete characterisation of the Mv virus responsible for the large measles outbreak in Milan and surrounding areas (Lombardy, Italy), which is a highly populated area with nearly 4 million inhabitants. From 1 March to 31 December 2017, 321 measles cases were laboratory-confirmed, 162 were sporadic cases and 159 were related to 75 outbreaks. The phylogenetic analysis enables us to establish whether links exist between concurrent measles cases. The aims of this study were to describe the genetic profile of circulating viruses and to track the pathway of measles transmission.

## Methods

### Mv amplification and sequencing

Total RNA was extracted from urine and oral fluid using the NucliSENS® easyMAG™ automated platform (bioMérieux bv, Lyon, France) according to the off-board lysis protocol. The amplification of a highly variable 450 nt sequence encoding the C-terminal part of the N gene (N-450) was performed [6, 8]. Reverse transcription with *Random Hexamer Primer* (Promega, Madison, WI, USA) using M-MLV RT Reverse transcriptase kit protocol (Promega, Madison, WI, USA) was performed. The N-450 fragment was amplified by semi-nested PCR using an equimolar mixture of Mv-specific primers with the Go Taq® DNA Polymerase (Promega, Madison, WI, USA). In the first step MN-F1 (5′-CCTGCTCTTGGACTGCATGAA-3′, nt. 951–971 of Mv strain Edmonston (Zagreb vaccine, Accession Number: AF266290.1)) and MV-B1 (5′-AACAATGATGGAGGGTAGGCG-3′, nt. 1629–1609) primers amplified a region of 678 bp, while the second step amplified a fragment of 644 bp selected by MV-F1 (5′-TACCCTCTGCTCTGGAGCTATGCC-3′, nt. 985–1008) and MV-B1 primers [14]. The first step was carried out under the following amplification reaction conditions: 5 min denaturation at 94 °C followed by 35 cycles of amplification. Each cycle consisted in a denaturation step at 94 °C for 30 s, an annealing step at 56 °C for 30 s and an elongation step at 72 °C for 45 s. The last cycle was followed by a 7 min elongation step at 72 °C. The second step involved 5 min denaturation at 94 °C followed by 35 cycles of amplification consisting in a denaturation step at 94 °C for 30 s, an annealing step at 60 °C for 30 s and an elongation step at 72 °C for 45 s. The last cycle was followed by a 7 min elongation step at 72 °C.

RT-PCR products were purified with the NucleoSpin*® Gel and PCR Clean-Up* (Macherey-Nagel GmbH & Co. KG, Germany) and nucleotide sequences were obtained by automated DNA sequencing based on fluorescent dye terminator on genetic analyser ABI PRISM 3100 Genetic Analyser (Applied Biosystem, Thermo Fisher, USA).

### Phylogenetic analysis

N-450 nucleotide sequences were analysed using the *Basic Local Alignment Search Tool*-*nucleotide* (*BLAST-n)* in order to identify similarities with previously reported strains [15].

Virus genotypes were designated according to the official WHO nomenclature and the sequences were submitted to the MeaNS database [8, 9].

Genotype as well as variant assignments were based on a Bayesian phylogenetic analysis. The Mv genotype A strain ‘MVs/Vaccine/Edmonston/’ was used as an outgroup.

Nucleotide sequences were aligned using Clustal X [16] and if necessary the alignments were manually edited using BioEDIT software [17]. A Bayesian phylogenetic tree was constructed with the best-fitting substitution model (Hasegawa–Kishino–Yano, HKY) chosen by JModelTest v. 2.1.7 [18] (http://darwin.uvigo.es/software/jmodeltest.html) using MrBayes [19]. A Markov chain Monte Carlo (MCMC) search was carried out for 5 × 10^6^ generations using tree sampling every 100^th^ generation and a burn-in fraction of 50%. Statistical support for specific clades was obtained by calculating the posterior probability (pp) of each monophyletic clade, and a posterior consensus tree was generated after a 50% burn-in. The tree was visualised and edited using Figtree software v 1.4.1.

### Classification of epidemic events

Descriptive data on measles cases were obtained from the Lombardy Region database. According to the national surveillance guidelines, the Lombardy Region Surveillance System requires physicians to report all suspected measles cases to the Local Health Units (LHUs) within 12 h from the onset of symptoms. The LHUs are responsible for carrying out all epidemiological investigations and collecting specimens from each suspected case. The personal data, clinical details and epidemiological status of each notified case were systematically reported to the Lombardy Region database. The data obtained were used to distinguish between sporadic cases or outbreaks and between indigenous, imported or imported-related cases.

We matched the epidemiological data with the genetic profile of the strains and the following definitions were used to classify the epidemic events:

*Transmission pathways*, events involving the continuous transmission of the same virus variant (autochthonous or imported) in a selected geographic area in a limited period;

*Autochthonous variant*, group of viruses with identical or nearly identical N-450 nucleotide sequences present in a selected geographic area and identified in non-imported cases;

*Imported variant*, group of viruses with identical or nearly identical N-450 nucleotide sequences previously not identified in a selected geographic area and identified in imported or import-related cases.

## Results

### Genotyping and phylogenetic analyses

Two-hundred and ninety-nine out of 321 (93.1%) confirmed cases (163 were classified as sporadic and 159 clustered in 75 different outbreaks) were successfully genotyped by sequencing the N-450 region. The BLAST-n analysis revealed that the sequences belonged to genotypes D8 and B3. The most frequently detected genotype was D8 (248/299, 82.9%).

**Fig. 1.**
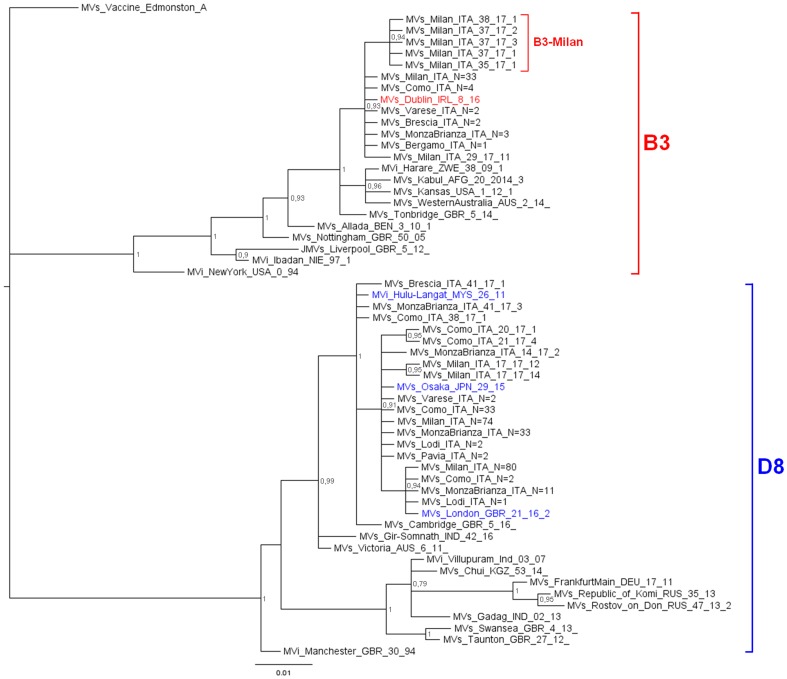
Phylogenetic analysis of the Mv-sequences identified in Lombardy during 2017. The evolutionary history of 299 N-450 Italian sequences was inferred with MrBayes software using the Bayesian Markov chain Monte Carlo (MCMC) method and Hasegawa–Kishino–Yano (HKY) model. Posterior probabilities of clades are indicated next to the nodes. The WHO named strains related to Italian sequences are coloured (red for B3, blue for D8). Genotypes and novel variant are indicated by squared parenthesis. All sequences presenting 100% identity and obtained from the same city or geographical area were considered as one single sequence which report in the label the number of total strains.

The phylogenetic analysis (Fig. 1) showed that 242 D8 sequences belonged to three WHO named strains: Osaka (Mv/Osaka.JPN/29.15, *N* = 146), London (Mvs/London.GBR/21.16/2, *N* = 94), Hulu Langat (MVi/Hulu-Langat.MYS/26.11, *N* = 2). For the remaining six sequences, five were closely related to the named strain Osaka and one to the named strain Hulu Langat.

D8 sequences belonging to the named strain Osaka and those related to this named strain, grouped significantly into a main variant D8-Osaka (pp = 0.91) (similarity range: 99.8–100%). Moreover, the D8-London is a named strain firstly identified in London in May 2016 (Mvs/London.GBR/21.16/2) and then observed for the first time in Milan in May 2017 (MVs/Milan.ITA/22.17/2, pp = 0.94). D8 sequences belonging to the named strain Hulu Langat and that related to this named strain, grouped significantly outside the variant D8-Osaka (pp = 1) (similarity range: 99.8–100%).

The 51 B3 sequences were closely related (pp = 0.93): 45 belonged to the WHO named strain Dublin (MVs/Dublin.IRL/8.16) and one was closely related to it (similarity: 99.8%). The remaining five sequences, temporarily called B3-Milan variant (MVs/Milan.ITA/35.17), grouped significantly (pp = 0.94) and were identical to each other.

### Pathways of transmission

**Fig. 2.**
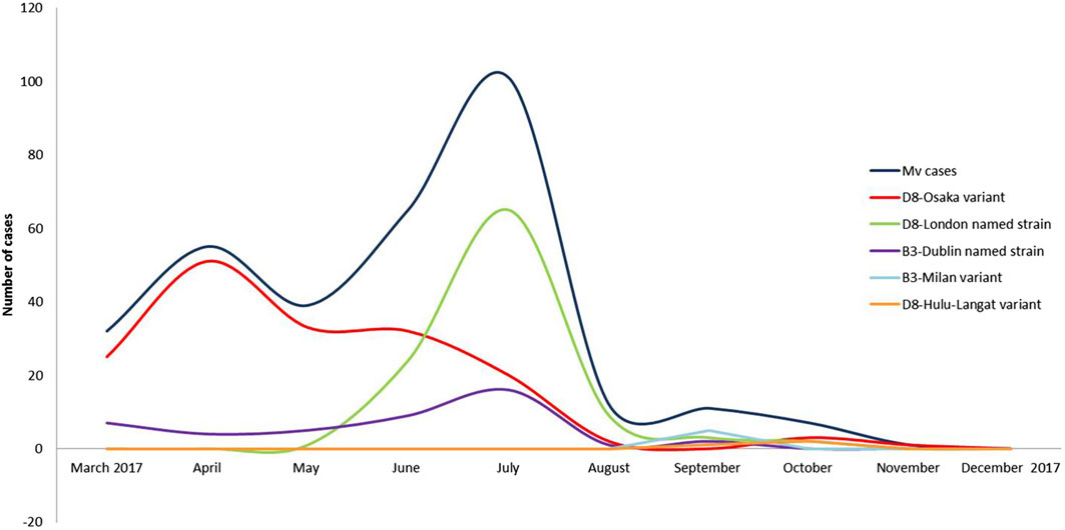
Epidemic curves of five different pathways of transmission sustained by five different variants from March to December 2017, in the surrounding areas and in the city of Milan.

Three events of continuous transmission of autochthonous variants (D8-Osaka, D8-London and B3-Milan variants) and two events of continuous transmission of imported variants (B3-Dublin and D8-Hulu Langat) tracked five different transmission pathways. These pathways defined two epidemic peaks: the first in April and the second in July 2017 (Fig. 2).

#### The D8-Osaka variant

The D8-Osaka was the prevalent variant identified in 146 autochthonous cases. In particular, 58 were notified as sporadic cases while 88 were involved in 47 outbreaks.

Events of continuous transmission of D8-Osaka were observed from March (MVs/Milan.ITA/09.17) to November (MVs/MonzaBrianza.ITA/47.17) 2017. Initially, the variant spread within the community and later spread in hospital settings. In March 2017, a serious outbreak affected three family members causing the death of one of them. The D8-Osaka was isolated from the biological samples of one of these cases. In June 2017, the same variant infected a transplant patient who developed late neurological sequelae and died in January 2018.

#### D8-London named strain

This previously undetected variant was identified in 94 autochthonous cases, 59 were notified as sporadic cases and 35 were related to 20 outbreaks. Events involving the continuous transmission of this strain were detected from June (MVs/Milan.ITA/22.17/2) to October (MVs/MonzaBrianza.ITA/41.17) 2017. The named strain was mainly spread in hospital settings.

#### B3-Dublin named strain

This strain was detected in 45 cases, 27 were notified as sporadic cases while 18 cases were related to nine outbreaks. Five cases out of 45 were imported, one case was imported-related and 39 cases were autochthonous.

The B3-Dublin was imported during March/April 2017 from various European countries (France, Romania) and Italian Regions (Apulia, Lazio, Piedmont), and successively caused imported-related outbreaks and then became established in the area, causing autochthonous cases. Events of continuous transmission of this variant peaked in July 2017, due to several nosocomial outbreaks. The last identification of the B3-Dublin was in September 2017 (MVs/Milan.ITA/36.17/3).

#### D8-Hulu-Langat variant

This variant was detected in two sporadic cases, of which one was notified as imported. The D8-Hulu-Langat strain was imported from South Africa in September 2017 (MVs/Como.ITA/38.17); in October 2017 this variant was detected in one case (MVs/MonzaBrianza.ITA/41.17/2) that was not epidemiologically travel-associated since the source of infection remains unknown.

#### B3-Milan variant

This previously undetected variant (MVs/Milan.ITA/35.17) was identified in five autochthonous cases, three cases were involved in a family outbreak and two sporadic cases. This variant was only observed in September 2017.

## Conclusions

A large measles epidemic occurred in Milan in 2017. In this study, we reconstructed the transmission pathways by matching the genetic profile of circulating Mv strains with epidemiological data.

Two different genotypes were identified: D8 and B3. The time of arrival of the D8 genotype in the area under study was estimated and dates back to March 1987 [20], while the B3 genotype was detected for the first time in Italy in the years 2006–2007 [21].

The phylogenetic analysis identified five different clusters: three belonging to the D8 genotype (D8-Osaka variant, D8-London named strain and D8-Hulu Langat variant) and two of the B3 genotype (B3-Dublin named strain and B3-Milan variant).

Two epidemic peaks were observed in April and July 2017. The first peak was mainly caused by the autochthonous D8-Osaka named strain. This named strain was described for the first time in Osaka, Japan in 2015 and was detected in Europe from 2016 onwards (in Germany, the Netherland and Switzerland). At the beginning of 2017, the D8-Osaka named strain was identified in several Italian Regions (Lazio, Piedmont, Emilia Romagna, Tuscany and Lombardy). It was the most prevalent in Milan and the surrounding areas and caused severe clinical courses and deaths. Several D8-Osaka cases were notified as sporadic cases, however the genetic profile suggested that it was a single large outbreak.

The second epidemic peak was characterised by the co-circulation of two different variants, the D8-London and the B3-Dublin named strain. The D8-London caused the highest number of cases, all were autochthonous cases. This named strain, which is phylogenetically related to the D8-Osaka variant, was observed for the first time in Milan in May 2017. A few weeks later, it caused an epidemic event in Finland where it was imported by an Italian student [22]. The D8-London named strain has caused events of long-lasting continuous transmission (June–September 2017) in Milan where it was disproportionally amplified by five nosocomial outbreaks.

The B3-Dublin caused the important outbreak in Romania in 2016–2017. In 2017, this named strain spread across all of the European countries [23]. In Milan, multiple introductions of this named strain were observed from March 2017 when it became established in the area thus replacing the B3-Como named strain which had circulated in Northern Italy from August 2015 onwards [24]. At the time of writing there is an ongoing measles outbreak of the B3-Dublin in the study area and this strain is considered to be endemic (persisting for more than 12 months [4]).

The genetic identification of circulating viruses enabled us to highlight the simultaneous occurrence of three different outbreaks caused by three variants (autochthonous or imported) in Milan and the surrounding area.

It is important to note that the D8-Osaka, D8-London and B3-Dublin named strains were associated with 12 nosocomial outbreaks which probably facilitated virus spread and the amplification of the virus in the community setting [25, 26]. The genetic analysis of the N-450 is a useful tool for establishing whether connections exist between concurrent measles cases [27].

Genotyping, phylogenetic analysis and the study of the epidemic curves could be considered important tools to follow the progress towards elimination. In fact, this multiple approach enables us to track the introduction of imported strains, to observe their persistence in a defined geographic area and highlight the occurrence of large epidemics and their periodic patterns.
